# Nitrincola salilacus sp. nov., isolated from Niya Fish Salt Lake Sediment in Xinjiang, Northwest China

**DOI:** 10.1099/ijsem.0.006894

**Published:** 2025-08-28

**Authors:** Qingyu Wang, Yi Xiao, Danning Gao, Longyi Su, Yongliang Yan, Yuhua Zhan, Zhengfu Zhou, Xiubin Ke

**Affiliations:** 1 Biotechnology Research Institute/Key Laboratory of Agricultural Microbiome (MARA), Chinese Academy of Agricultural Sciences, Beijing 100081, PR China

**Keywords:** chemotaxonomic characterization, comparative genomics, genomic analysis, *Nitrincola*, salt lake

## Abstract

A Gram-stain-negative, aerobic, rod-shaped, oxidase- and catalase-positive and non-motile bacterium, named MINF-07-Sa-05^T^, was isolated from sediment samples collected from Niya Fish Salt Lake in Xinjiang, northwest China. Phylogenetic tree based on 16S rRNA gene sequences showed that strain MINF-07-Sa-05^T^ consistently fell within the genus *Nitrincola* and formed a clade together with *Nitrincola alkalilacustris* ZV-19^T^ (98.97%) and *Nitrincola lacisaponensis* 4CA^T^ (96.16%). Genomic average nucleotide identity values for strain MINF-07-Sa-05^T^ with the type strains within the genus *Nitrincola* ranged from 69.8 to 87.8%, while the *in silico* DNA–DNA hybridization values for strain MINF-07-Sa-05^T^ with these strains ranged from 20.4 to 33.7%. The genome sequence size of the strain was 4.81 Mb, with a DNA G+C content of 52.6 mol%. Its growth occurred at 4–40 °C, at pH 6.0–10.0, and tolerated up to 9% (w/v) NaCl. The major fatty acids contained summed feature 8 (C_18:1_* ω*7*c*/C_18:1_* ω*6*c*; 59.8%), summed feature 3 (C_16:1_* ω*7*c*/C_16:1_* ω*6*c*; 20.4%) and C_16:0_ (13.8%). The primary respiratory quinone was ubiquinone-8. The main polar lipids were phosphatidylglycerol and phosphatidylethanolamine. Genomic analyses showed a high abundance of genes involved in the nitrogen, sulphur cycle and antibiotic resistance. According to the genotypic, phylogenetic and chemotaxonomic data, strain MINF-07-Sa-05^T^ represents a novel species within the genus *Nitrincola*, for which the name *Nitrincola salilacus* sp. nov. is proposed. The type strain is MINF-07-Sa-05^T^ (=GDMCC 1.4420^T^=JCM 37233^T^).

## Data Summary

The authors confirm all supporting data, code and protocols have been provided within the article or through supplementary data files.

## Introduction

The genus *Nitrincola* [1], belonging to the order *Oceanospirillales* [2] and family *Oceanospirillaceae* within phylum *Pseudomonadota*, class *Gammaproteobacteria*, was initially proposed by Dimitriu *et al*. in 2005 [1]. Currently, the genus *Nitrincola* comprised eight validly published species, including *Nitrincola alkalilacustris* ZV-19^T^ (doi:10.1099/ijsem.0.002437) [3], *Nitrincola schmidtii* R4-8^T^ (doi:10.1099/ijsem.0.002437) [3]*, Nitrincola lacisaponensis* 4CA^T^ (doi:10.1099/ijs.0.63647–0) [1], *Nitrincola alkalisediminis* MEB087^T^ (doi:10.1099/ijsem.0.000868) [4], *Nitrincola tibetensis* xg18^T^ (doi:10.1099/ijsem.0.003111) [5], *Nitrincola iocasae* KXZD1103^T^ (doi:10.1099/ijsem.0.004352) [6], *Nitrincola tapanii* MEB193^T^ (doi:10.1099/ijsem.0.003883) [7] and *Nitrincola nitratireducens* AK23 (doi:10.1016 /j.syapm.2015.09.002) [8]. *N. lacisaponensis* 4CA^T^ [1] was isolated from Soap Lake, a saline-alkaline meromictic lake in the USA. *N. alkalilacustris* ZV-19^T^ was isolated from soda pans situated in Hungary’s Kiskunság National Park [3]. *N. alkalisediminis* MEB087^T^ and *N. tapanii* MEB193^T^ were isolated from both sediment and water samples collected from Lonar Lake, an Indian soda lake exhibiting alkaline saline conditions in the Buldhana district of Maharashtra [4,7]. All strains of the genus *Nitrincola* were isolated from saline-alkaline environments and were alkaliphilic, exhibiting salt tolerance [1,8]. A polyphasic taxonomic study of strain MINF-07-Sa-05^T^ and reference strains was performed by phylogenetic and phenotypic characterization. Furthermore, the whole-genome sequencing of the MINF-07-Sa-05^T^ strain and comparative genomic analysis of nine strains of the genus *Nitrincola* were provided to explain the potential ecological function. Our results indicated that strain MINF-07-Sa-05^T^ represented a novel species of the genus *Nitrincola*, for which we proposed the species name *Nitrincola salilacus* sp. nov.

## Methods

### Isolation and culture conditions

Strain MINF-07-Sa-05^T^ was isolated from sediment samples collected from Niya Fish Salt Lake under the reeds in Xinjiang, northwest China (37°23′21′′N, 82°97′79′′E). The sediment samples were diluted and cultured on 2216E plates at 30 °C for 48 h, after which well-isolated colonies were picked and serially streaked onto fresh 2216E plates incubated at 30 °C to obtain single colonies. Strain MINF-07-Sa-05^T^ was obtained after several re-streaking and transfer onto 2216E plates. The pure culture of strain MINF-07-Sa-05^T^ was preserved at −80 °C in 2216E medium with 25% (v/v) glycerol. The phylogenetically related type strains were used as reference strains. *N. lacisaponensis* 4CA^T^ was obtained from the Deutsche Sammlung von Mikroorganismen und Zellkulturen GmbH (DSMZ) and *N. alkalisediminis* MEB087^T^ was obtained from the Japan Collection of Microorganisms (JCM).

### Phylogenetic analysis

Genomic DNA was extracted from strain MINF-07-Sa-05^T^ using the Bacteria DNA Kit (Tiangen Biotech, China). The 16S rRNA genes were amplified by PCR with the primers 27F (5′-AGAGTTTGATCCTGGCTCAG-3′) and 1492R (5′-GGTTACCTTGTTACGACTT-3′) [9]. The 16S rRNA gene sequence was obtained, and similarity with other microbial sequences was determined using the EzBioCloud server (www.ezbiocloud.net/) [10]. The 16S rRNA gene sequences of strain MINF-07-Sa-05^T^ and related taxa were aligned using clustal x (version 2.0) [11]. Phylogenetic trees were reconstructed using mega11 [12] with the neighbour-joining (NJ) [13], minimum evolution (ME) [14] and maximum likelihood (ML) [15] algorithms, respectively. Distances were calculated according to the Kimura two-parameter model [16]. A genome phylogenetic tree based on orthologous proteins of the genus *Nitrincola* was constructed by FastTree [17] according to the ML method. The topology of the phylogenetic tree was estimated by bootstrap analysis of 1,000 replicates [15]. The average nucleotide identity (ANI) calculator [18] (www.ezbiocloud.net/tools/ani) and the Genome-to-Genome Distance Calculator (v. 2.1) [19] (http://ggdc.dsmz.de/home.php) were used to calculate the ANI and digital DNA–DNA hybridization values, respectively.

### Phenotypic and biochemical characteristics

Cell morphology was examined using a light microscope (Nikon, 80i, Tokyo, Japan) and transmission electron microscope (Hitachi 7500, Tokyo, Japan) after incubation on 2216E agar plates for 48 h at 30 °C. The temperature range for growth was determined by incubating strain MINF-07-Sa-05^T^ at 0, 4, 10, 15, 20, 25, 30, 35, 37, 40, 45 and 50 °C. The pH value range was determined in 2216E with pH values of 4.0–12.0 at intervals of 1 pH unit. The optimal concentration of NaCl for growth was investigated using NaCl-free 2216E with different NaCl concentrations (0–11.5% at 0.5% increments, w/v). The pH growth range was tested at 30 °C in 2216E using KH_2_PO_4_/K_2_HPO_4_ or Na_2_CO_3_/NaHCO_3_ buffer system. Catalase and oxidase activities were detected by observing bubble production in 3% (v/v) H_2_O_2_ solution and colour variance of 1% (w/v) tetramethylp-phenylenediamine, respectively. Cell motility was examined using the hanging-drop technique [20]. Anaerobic growth was tested on 2216E agar with an anaerobic pouch (MGC, Mitsubishi) at 30 °C. Gram reaction was determined by using a Gram-staining kit (Huankai) and the KOH lysis method [21]. Additional physiological and biochemical tests were performed via API 20E and API 20NE strips (bioMérieux) according to the manufacturer’s instructions. Carbon source utilization and chemical sensitivity assays were performed via a Biolog GEN III MicroPlate™ (Biolog) following the manufacturer’s instructions. Hydrolysis of starch was performed as described by Gerhardt *et al*. [21].

### Chemotaxonomic analyses

Fatty acids from whole cells grown on 2216E at 30 °C for 48 h were saponified, methylated and extracted using the standard midi protocol (Sherlock Microbial Identification System, version 6.0B), and then analysed by gas chromatography (Agilent Technologies 6850) and identified using the TSBA6.0 database of the Microbial Identification System [22]. Polar lipids were extracted according to the procedures described by Minnikin *et al*. and subsequently separated using two-dimensional TLC [23,24] (silica gel, 10×10 cm; Merck) with chloroform/methanol/water (65:25:4, v/v/v) in the first direction, followed by chloroform/acetic acid/methanol/water (80:18:12:5, v/v/v/v) in the second direction. The components with functional groups were identified with molybdatophosphoric acid, ninhydrin, molybdenum blue, Dragendorff’s reagent and *α*-naphthol solution as described previously, respectively [23,24]. Respiratory quinones were extracted from the lyophilized cells and then analysed by using an HPLC (Agilent 1260) as described previously [25,26].

### Complete genome sequencing and analysis

The whole-genome sequencing of MINF-07-Sa-05^T^ was performed using the Illumina PE150 platform and PacBio Sequel system [27]. For the PacBio sequencing library, 5–10 µg genomic DNA was sheared into 10–15 Kb fragments using a g-TUBE device. The library was constructed using the SMRTbell® Express Template Preparation Kit 2.0. Briefly, the sheared fragments were through single-strand overhangs, removing DNA damage repair, end-repair, A-tailing and ligation of barcoded overhang adapters. The library was quantified using a Qubit 3.0 Fluorometer (Invitrogen, Carlsbad, CA), and the size of the library was checked using an Agilent 2100 Bioanalyzer System. Subsequent steps were followed as per the manufacturer’s instructions to prepare the SMRTbell library. The library was sequenced using the PacBio Sequel platform. PacBio reads were assembled using Hifiasm [28]/Canu [29]. And then, we recorrected the genome with the software Pilon using previous Illumina data. The Prodigal [30]/Augustus [31] gene-finding software had been used for finding coding genes. tRNAs were detected in the genome using the program tRNAscan-SE [32] with default parameter settings. rRNAs were identified by using Barrnap. Other RNAs were identified by the Rfam database. The coding genes were annotated with the National Center for Biotechnology Information nr database by diamond. Then, the functions of genes were annotated by the Gene Ontology (GO) [33] database, and the pathways were annotated using the Kyoto Encyclopedia of Genes and Genomes (KEGG) [34] database. The proteins encoded by genes were classified according to a phylogenetic classification by the database of Clusters of Orthologous Groups (COG) of proteins [35]. diamond was used to search the protein sequences with the CAZy database, Swiss_Prot database [36], Pfam database, CARD database, VFDB database or DFVF database with *E*<1e−5.

### Comparative genomics of *Nitrincola* species

The present study compared the genomes of nine bacteria belonging to the genus *Nitrincola*. The nine bacteria and GeneBank accession numbers were as follows: *N. alkalilacustris* ZV-19, GCA_008973755.1; *N. lacisaponensis* 4CA, GCA_000691225.1; *N. tapanii* MEB193, GCA_008368715.1; *N. iocasae* KXZD1103, GCA_008727795.1; *N. alkalisediminis* MEB087, GCA_009821115.1; *N. tibetensis* xg18, GCA_003284585.1; *N. schmidtii* R4-8, GCA_008973715.1; *N. nitratireducens* AK23, GCA_000585235.1; and *N. salilacus* MINF-07-Sa-05, GCA_046271865.1. The Bacterial Pan Genome Analysis (BPGA) pipeline [37] was used for the pan genome analyses, as described previously [38]. In brief, the clustering tool USEARCH was used to cluster protein families. A 50% sequence identity was considered as the cut-off value for orthologous clustering to obtain the pan and core genomes. After obtaining the core genome of the genus *Nitrincola*, the OrthoFinder [39,40] was used to perform an all-versus-all blast search and identify clusters of orthologous genes (OGs), and those OGs were then aligned and concatenated by muscle [41]. Synteny analysis of the genomes with their closest related strain types was conducted using the progressive Mauve tool [42].

## Results

### Phylogenetic analysis

**Fig. 1.**
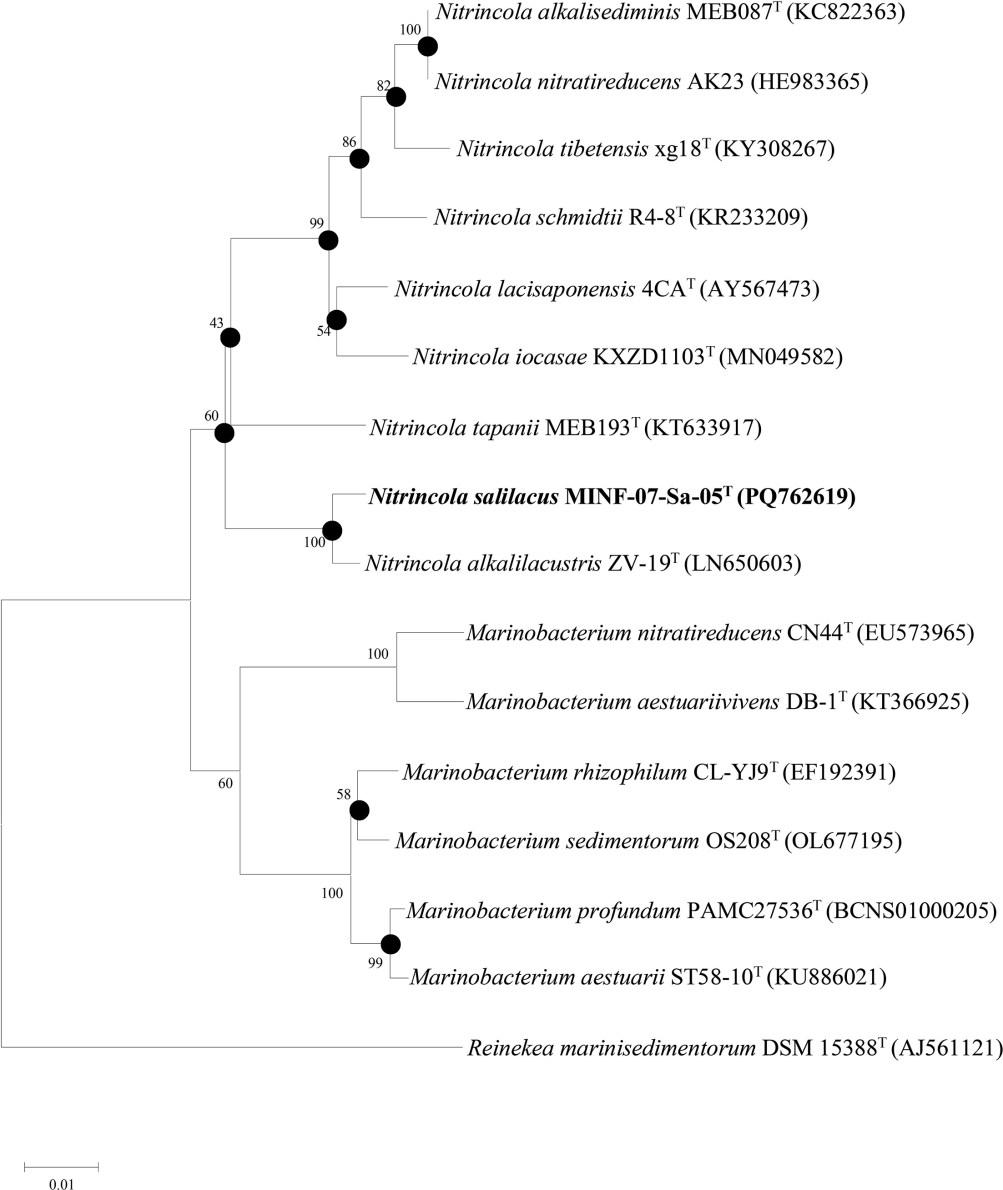
NJ tree based on 16S rRNA gene sequences showing the relationships between strain MINF-07-Sa-05^T^ and other related taxa in the family of *Oceanospirillaceae*. Bootstrap values were expressed as a percentage of 1,000 replications. Only bootstrap values of more than 50% were shown. *Reinekea marinisedimentorum* DSM 15388^T^ was used as an outgroup. The GenBank accession numbers were provided after the species names. Bar, 0.01 substitutions per nucleotide position.

**Fig. 2.**
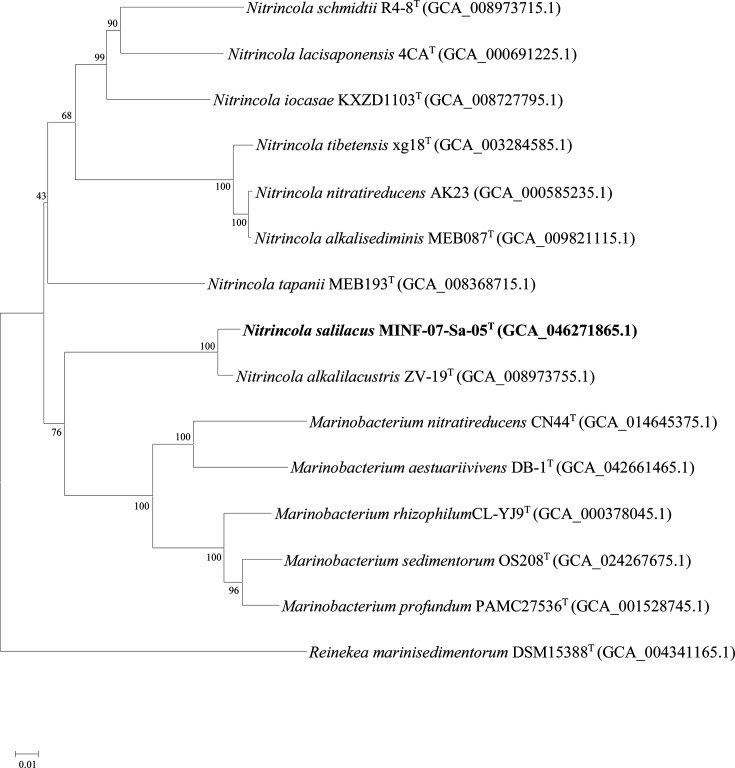
Phylogenetic tree based on the whole-genome sequences showing the relationships between strain MINF-07-Sa-05^T^ and other related taxa in the family of *Oceanospirillaceae. Reinekea marinisedimentorum* DSM 15388^T^ was used as an outgroup. GenBank accession number of each strain was in parentheses.

Strain MINF-07-Sa-05^T^ exhibited 16S rRNA gene sequence similarity of 98.97% to *N. alkalilacustris* ZV-19^T^, 96.2% to *N. lacisaponensis* 4CA^T^, 96.0% to *N. tapanii* MEB193^T^ and 95.20% to *N. alkalisediminis* MEB087^T^. Phylogenetic analysis was performed using the NJ method with Kimura two-parameter as a model of nucleotide substitution. In the NJ phylogenetic tree (Fig. 1), strain MINF-07-Sa-05^T^ clustered with the clade consisting of *N. alkalilacustris* ZV-19^T^. The topologies of the ME (Fig. S1, available in the online Supplementary Material) and ML (Fig. S2) trees were highly similar to that of the NJ tree. The genome phylogenetic analysis revealed that strains MINF-07-Sa-05^T^ and *N*. *alkalilacustris* ZV-19^T^ grouped together within the same clade, suggesting a close evolutionary relationship (Fig. 2). The genomic ANI values of strain MINF-07-Sa-05^T^ with the type strains of the genus *Nitrincola* ranged from 69.8 to 87.% (Table S1), which were below the threshold value for delineating species (95–96%) [43]. The *in silico* DDH values of strain MINF-07-Sa-05^T^ with the type strains of the genus *Nitrincola* ranged from 20.4 to 33.7% (Table S1), which were below the standard accepted cut-off value (70%) [44].

These phylogenetic data demonstrated that strain MINF-07-Sa-05^T^ should be assigned to the genus *Nitrincola*, family *Oceanospirillaceae*.

### Phenotypic and biochemical characteristics

**Fig. 3.**
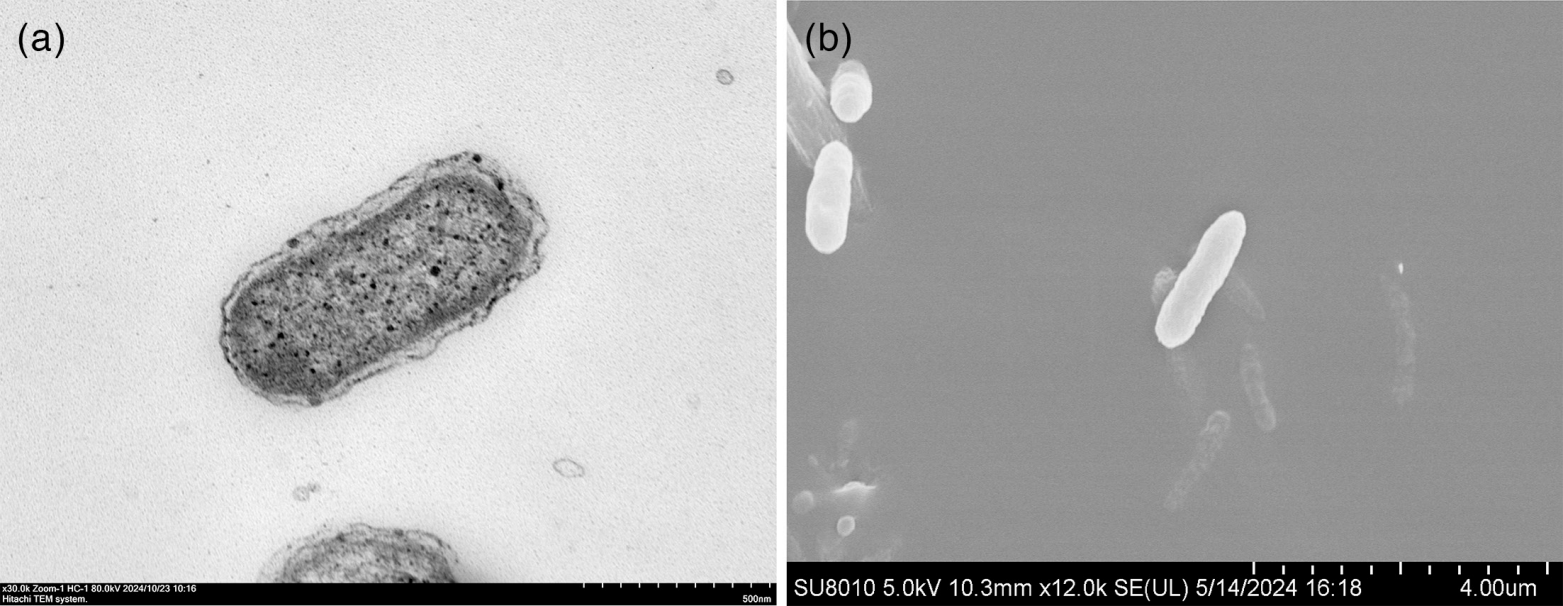
(**a**) Transmission electron micrograph of strain MINF-07-Sa-05^T^. Bar, 500 nm. (**b**) Scanning electron micrograph of cells. Bar, 4.0 µm.

Cells of strain MINF-07-Sa-05^T^ were Gram-stain-negative, rod-shaped (0.3–0.5 µm wide and 1.4–2.7 µm long) (Table 1), aerobic, non-motile (Figs 3 and S3) and oxidase- and catalase-positive. Strain MINF-07-Sa-05^T^ formed circular, entire, beige, smooth and convex colonies on the modified 2216E medium after 48 h of incubation at 30 °C. Starch was not hydrolysed. The phenotypic characteristics of strain MINF-07-Sa-05^T^ were reported in the species description (Table 1). Growth of strain MINF-07-Sa-05^T^ occurred at 4–40 °C (optimum, 25–35 °C), pH 6.0–10.0 (optimum, pH 7.0–9.0) and with 0–9.0% (w/v) NaCl (optimum, 3.5–4.5%). Other differences in physiological characteristics with respect to related species of the genus *Nitrincola* were listed in Table 1. All the tests based on API 20E microplates produced negative results. In API 20NE rests, positive responses were observed for aesculin hydrolysis and nitrate reduction; other tests produced negative results. In the Biolog GEN III system, the cells were positive for sucrose, d-turanose, *α*-d-glucose, d-fructose, d-galactose, d-mannitol, myo-inositol, l-alanine, l-arginine, l-aspartic acid, l-serine, pectin, d-galacturonic acid, l-galactonic acid lactone, d-gluconic acid, citric acid, *α*-keto-glutaric acid, d-malic acid, l-malic acid, Tween 40, *β*-hydroxy-d, l-butyric acid and acetoacetic acid. Other tests based on Biolog GEN III microplates produced negative or weak results.

**Table 1.** Comparative characteristics of strain MINF-07-Sa-05^T^ with the four reference strains Strains: 1, MINF-07-Sa-05^T^; 2, *N. alkalilacustris* ZV-19^T^; 3, *N. lacisaponensis* 4CA^T^; 4, *N. tapanii* MEB193^T^; 5, *N. alkalisediminis* MEB087^T^. +, Positive; −, negative; w, weakly positive reaction. All data were obtained from this study unless otherwise indicated.

Characteristic	1	2*	3	4†	5
Cell length (μm)	1.4–2.7	0.8–1.5	1.3–2.3	0.9–1.6	1.4–2.2
Cell width (μm)	0.3–0.5	0.5–0.8	0.3–0.4	0.2–0.6	0.7–1.17
Motility	Non-motile	Motile	Motile	Motile	Non-motile
Temperature range (optimum), °C	4–40 (25–35)	10–37 (30)	4–40 (25–35)	10–37 (30)	4–37 (25–30）
pH range (optimum)	6–10 (7–9)	8–11 (9–10)	7–11 (7–9)	8–10.5 (9.0)	6–11 (7–9）
NaCl range (optimum), % (w/v)	0–9 (3.5–4.5)	0–8 (0–5)	0–10 (4–5.5)	0–6 (1)	0–10 (4–6）
Major polar lipids	PE, PG	PE, PG, PS	PE, DPG, PG	PG, DPG	PE, DPG, PC
DNA G+C content (mol%)	52.6	54.5	52.1	50.8	49.3
Oxidation of (Biolog GEN III)					
Gentiobiose	w	−	+	−	−
Sucrose	+	−	−	−	w
d-Turanose	+	−	−	−	w
d-Melibiose	w	−	+	−	−
d-Mannose	w	−	+	−	−
3-Methyl glucose	w	−	+	w	−
d-Fucose	w	w	+	w	−
l-Fucose	w	−	+	+	−
d-Sorbitol	w	−	−	−	+
d-Mannitol	+	−	−	−	+
d-Arabitol	w	−	−	−	w
myo-Inositol	+	−	−	−	−
l-Alanine	+	−	−	−	+
l-Arginine	+	−	−	−	−
l-Aspartic acid	+	w	−	−	−
l-Serine	+	−	−	−	−
Pectin	+	−	−	−	w
l-Galactonic acid lactone	+	−	w	−	−
d-Gluconic acid	+	−	−	−	−
d-Glucuronic acid	w	−	+	+	w
Glucuronamide	−	w	+	+	w

* Data from [1]. †Data from [7].

### Chemotaxonomic characteristics

The cellular fatty acid profiles of strain MINF-07-Sa-05^T^ and the other four type strains of the genus *Nitrincola* were simultaneously analysed in this study. The major fatty acids (>10% of total fatty acids) in strain MINF-07-Sa-05^T^ were summed feature 8 (C_18:1_* ω*7*c*/C_18:1_* ω*6*c*; 59.8%), summed feature 3 (C_16:1_* ω*7*c*/C_16:1_* ω*6*c*; 20.4%) and C_16:0_ (13.8%) (Table 2). This profile was similar to the other four type strains, with differences in the proportions of some fatty acids. The polar lipids of strain MINF-07-Sa-05^T^ contained phosphatidylglycerol, phosphatidylethanolamine, an unidentified aminolipid, an unidentified phospholipid, unidentified glycolipids and an unidentified lipid (Fig. S4). Additionally, the predominant quinone detected in strain MINF-07-Sa-05^T^ was ubiquinone-8 (Q-8), consistent with other type strains of the genus *Nitrincola*, except ubiquinone-9 (Q-9, 1%) was also present only in *N. schmidtii* R4-8^T^.

**Table 2.** Fatty acid composition (expressed as percentage) of strain MINF-07-Sa-05^T^ and closely related reference strains of the genus *Nitrincola* Strains: 1, MINF-07-Sa-05^T^; 2, *N. alkalilacustris* ZV-19^T^; 3, *N. lacisaponensis* 4CA^T^; 4, *N. tapanii* MEB193^T^; 5, *N. alkalisediminis* MEB087^T^.

Fatty acid	1	2†	3	4‡	5
C_10:0_	0.8	tr	3.8	2.6	2.4
C_10:0_ 3-OH	2.8	2.8	6.6	4.7	4.6
C_12:0_	0.8	1.5	2.8	2.6	2.7
C_16:0_	13.8	13.6	13.0	24.6	16.3
C_18:0_	1.1	1.3	tr	1.5	tr
*Summed feature 3	20.4	13.8	13.3	9.9	18.5
*Summed feature 8	59.8	65.3	59.7	52.3	53.1

* Summed features were fatty acids that cannot be resolved reliably from other fatty acids using the chromatographic conditions chosen. The MIDI system grouped these fatty acids as one feature with a single percentage of the total: summed feature 3, C_16:1_* ω*7*c*/ C_16:1_* ω*6*c*; summed feature 8, C_18:1_* ω*7*c*/C_18:1_* ω*6*c*. Only fatty acids accounting for at least 0.5% of the total acid content in strain MINF-07-Sa-05T were listed. tr, trace (<0.5%). Values were the mean of three replicates. All data were obtained from this study unless otherwise indicated. †Data from [1]. ‡Data from [7].

### Complete genome sequencing and analysis

The complete genome sequence of strain MINF-07-Sa-05^T^ was 4,817,508 bp, with a DNA G+C content of 52.60 mol%. The genome contained 4,363,944 bp of coding regions, 94 RNA genes, 4,404 protein-coding genes with enzymes, 3,584 genes assigned to COGs, 1,876 COG clusters and 325 genes with signal peptides. A total of 4,236, 2,755, 3,192 and 3,584 proteins were assigned to NR, GO, KEGG and COG, respectively (Table S2). Based on this database, 20 genes associated with nitrogen cycling were identified (Table S3). The genes of nitrogen removal, including *narG* (nitrate reductase 1, alpha subunit), *napA* (periplasmic nitrate reductase subunit), *napB* (periplasmic nitrate reductase electron transfer subunit), *nirB* (nitrite reductase large subunit), *nirD* (nitrite reductase small subunit) and *nirK* (nitrite reductase), indicated its capability for denitrification. Additionally, a total of 19 gene markers of the sulphur cycling were retrieved from the genome of strain MINF-07-Sa-05^T^, which involved in 7 key sulphur cycling pathways, including assimilatory sulphate reduction, dissimilatory sulphur reduction and oxidation, sulphur reduction, sulphur oxidation system, sulphur oxidation, organic sulphur transformation and linkages between inorganic and organic sulphur transformation (Table S4). Among these, genes *cysD* and *cysN* participated in sulphate activation to adenosine 5′-phosphosulphate, and *cysC* converted adenosine 5′-phosphosulphate to phosphoadenosine 5′-phosphosulphate. The genes *cysN* and *cysC* encoded the bifunctional enzyme CysN/CysC complex, which catalysed sulphate activation to adenosine 5′-phosphosulphate; this was further converted to phosphoadenosine 5′-phosphosulphate by *cysD. CysH* encoded 5′-phosphosulphate to phosphoadenosine 5′-phosphosulphate reductase, reducing 5′-phosphosulphate to phosphoadenosine 5′-phosphosulphate to sulfite, while *cysI* and *cysJ* encoded sulfite reductase, catalysing sulfite reduction to sulfide. Furthermore, a total of 298 genes were associated with antibiotic resistance. One hundred sixty-two and 96 genes were related to antibiotic efflux and antibiotic target alteration, respectively (Fig. S5). From all detected antibiotic resistance genes, *macB* and *mtrA*, encoding a class of macrolide and *evgS*, encoding a fluoroquinolone class antibiotic resistance, could be found in the genomes of strain MINF-07-Sa-05^T^ (Table S5), indicating its potential tolerance to multiple classes of antibiotics.

### Comparative genomics of *Nitrincola* species

**Fig. 4.**
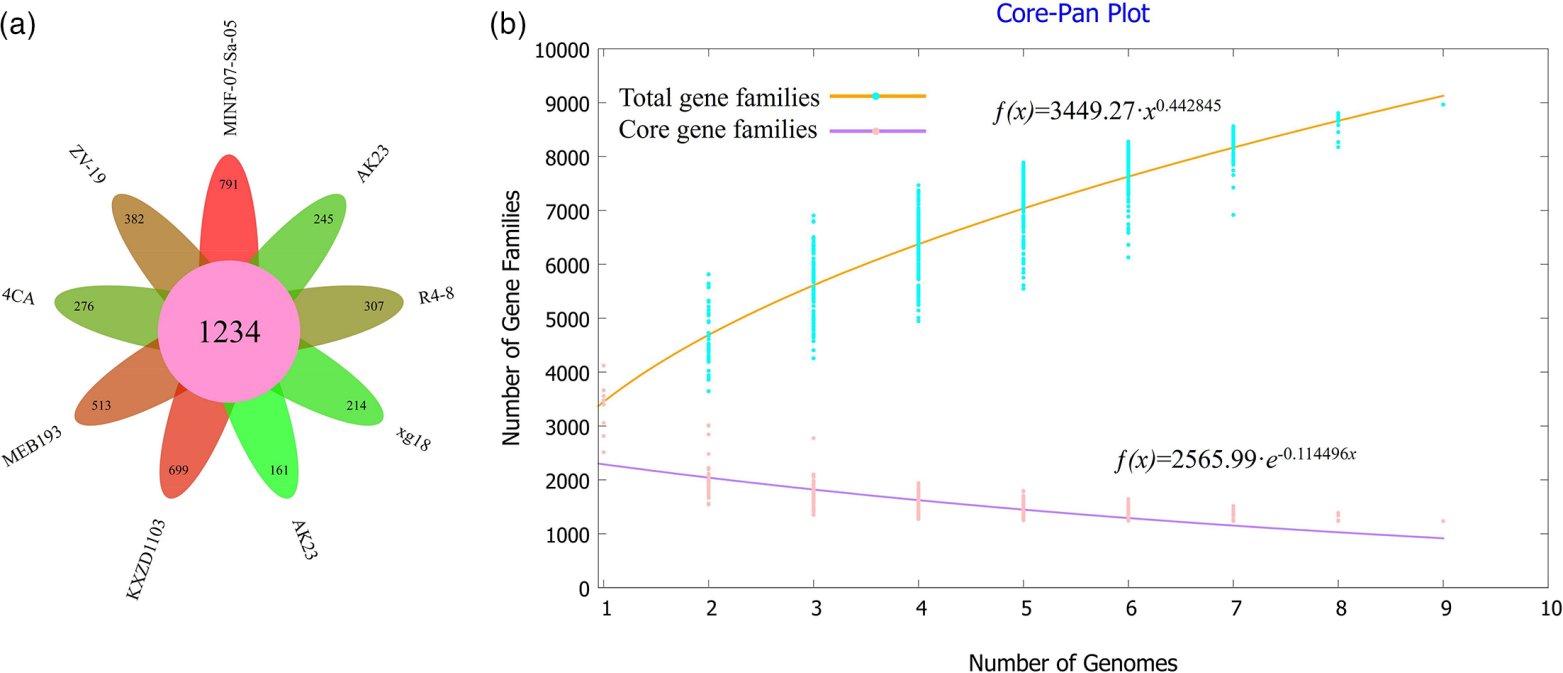
Pan-genomic analysis of genus *Nitrincola*. (**a**) The Venn diagram of core, dispensable and unique genes. Each strain was represented by a coloured oval. The centre was the number of orthologous coding sequences shared by strains (i.e. the core genome). Numbers in non-overlapping portions of each oval showed the numbers of CDSs unique to each strain. (**b**) Mathematical modelling of the pan genome and core genome of genus *Nitrincola*.

**Fig. 5.**
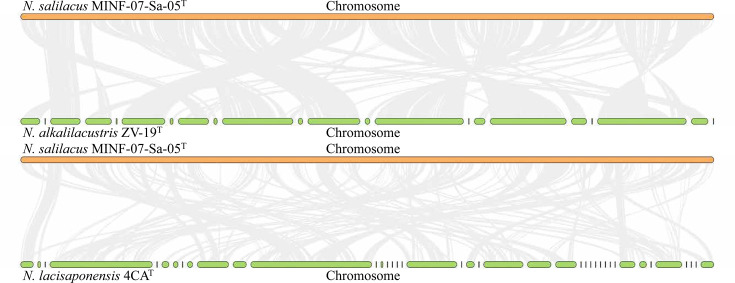
Genome alignment between strain MINF-07-Sa-05^T^ and two closely related type strains.

A summary of nine whole-genome comparisons between strain MINF-07-Sa-05^T^ and members of the genus *Nitrincola* (Table S6) was used for the comparative genomic analysis using the BPGA pipeline. The size and G+C content of the genomes used in this study ranged from 2.97 to 4.82 MB and 45.8–54.5 mol%, respectively. Generally, a local database containing 9 genomes and 31,107 putative protein-coding genes was created. Based on this database, 1,234 (27.82%) shared orthologous coding sequences were clustered into the core genome of *Nitrincola*, 2,096 (47.26%) were represented in the accessory genome and 791 (17.84%) were identified as strain-unique genes (Fig. 4a). Therefore, a highly reliable mathematical extrapolation of the pan and core genome was constructed (Fig. 4b). The total genes increased in the pan-genome of genus *Nitrincola* with the rise in the analysed genome number, suggesting that the pan-genome was open. Meanwhile, the number of core genes reached a stable level after six species were included in the analysis, indicating that the core genome of genus *Nitrincola* was highly conserved. Moreover, the linear comparison indicated that strain MINF-07-Sa-05^T^ shared more gene clusters with *N*. *alkalilacustris* ZV-19^T^ than with *N*. *lacisaponensis* 4CA^T^ and that gene segments had genome rearrangements that were inverted and translocated (Fig. 5).

Based on the results of phylogenetic, phenotypic, physiological and chemotaxonomic analyses, strain MINF-07-Sa-05^T^ is considered to represent a novel species, for which the name *Nitrincola salilacus* sp. nov. is proposed.

## Discussion

### Description of *Nitrincola salilacus* sp. nov.

*Nitrincola salilacus* sp. nov. (sa.li.la’ cus. L. masc. n. *sal*, salt; L. masc. n. *lacus*, lake; N.L. gen. n. *salilacus*, of a salt lake).

Cells were Gram-stain-negative, rod-shaped (~0.3–0.5 µm wide and 1.4–2.7 µm long), aerobic, non-motile and oxidase- and catalase-positive. Colonies were circular, entire, beige, smooth and convex on 2216E medium. Starch was not hydrolysed. Grew at 4–40℃, pH 6.0–10.0 and in the presence of 0–9% (w/v) NaCl, with optimum growth occurring at 25–35℃, pH 7.0–9.0 and with 3.5–4.5% (w/v) NaCl. All the tests based on API 20E microplates produced negative results. In API 20NE rests, positive responses were observed for aesculin hydrolysis and nitrate reduction; other tests produced negative results. In the Biolog GEN III system, positive for the utilization of sucrose, d-turanose, *α*-d-glucose, d-fructose, d-galactose, d-mannitol, myo-inositol, l-alanine, l-arginine, l-aspartic acid, l-serine, pectin, d-galacturonic acid, l-galactonic acid lactone, d-gluconic acid, citric acid, *α*-keto-glutaric acid, d-malic acid, l-malic acid, Tween 40, *β*-hydroxy-D, l-butyric acid and acetoacetic acid. Other tests based on Biolog GEN III microplates produced negative or weak results. The major cellular fatty acids were summed feature 8 (C_18:1_* ω*7*c*/C_18:1_* ω*6*c*), summed feature 3 (C_16:1_* ω*7*c*/C_16:1_* ω*6*c*) and C_16:0_. The major respiratory ubiquinone was Q-8, and the predominant polar lipids were phosphatidylglycerol and phosphatidylethanolamine. The genome of the type strain was characterized by a size of 4.81 Mb and a DNA G+C content of 52.6 mol%.

The type strain, MINF-07-Sa-05^T^ (=GDMCC 1.4420^T^=JCM 37233^T^), was isolated from sediment samples collected from Niya Fish Salt Lake in Xinjiang, Northwest China. The GenBank accession numbers of the 16S rRNA gene sequence and genome sequence of strain MINF-07-Sa-05^T^ were PQ762619 and GCA_046271865.1, respectively.

## Supplementary material

10.1099/ijsem.0.006894 Uncited Fig. S1.

## Abbreviations

ANI: average nucleotide identity
BPGA: Bacterial Pan Genome Analysis
COG: Clusters of Orthologous Groups
GO: gene ontology
KEGG: Kyoto Encyclopedia of Genes and Genomes
ME: minimum evolution
ML: maximum likelihood
NJ: neighbour-joining
OGs: orthologous genes
