# Supplementary material for: Nitrincola salilacus sp. nov., isolated from Niya Fish Salt Lake Sediment in Xinjiang, Northwest China
Source: Int J Syst Evol Microbiol. 2025 Aug 28;75(8):006894. doi: 10.1099/ijsem.0.006894 (PMC12394749; doi:10.1099/ijsem.0.006894)
Supplement: Uncited Fig. S1. [file ijsem-75-06894-s001.pdf]

Supplementary Material

Nitrincola salilacus sp. nov., isolated from Niya Fish Salt Lake Sediment in Xinjiang, Northwest China

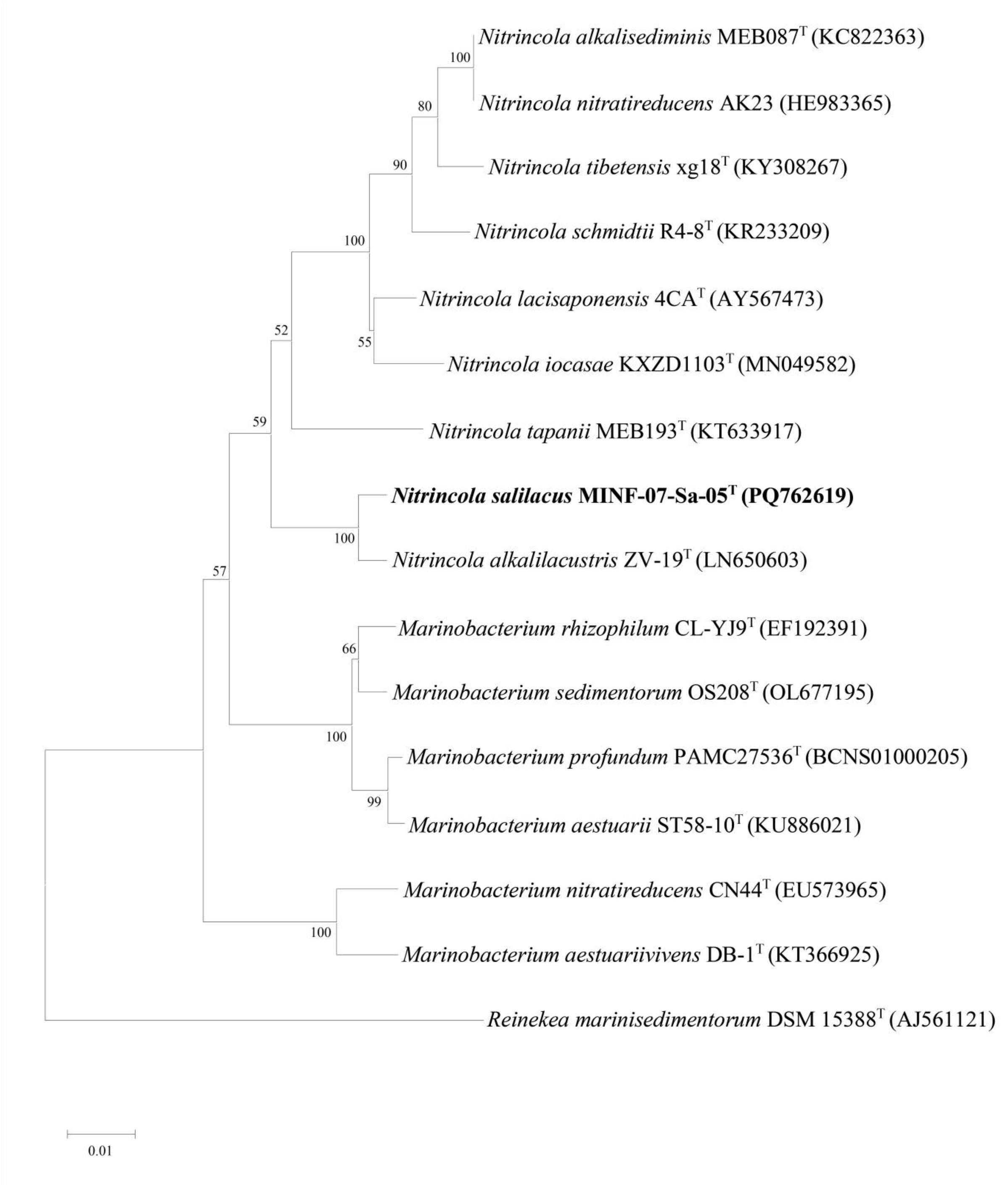

Fig. S1. Minimum-evolution phylogenetic tree based on 16S rRNA gene sequences showing the relationships between strain MINF-07-Sa-05^T and other related taxa in the family of *Oceanospirillaceae*. Bootstrap values were expressed as a percentage of 1000 replications.

Company Limited by Guarantee l Registered in England No. 1039582 l Registered Office Charles Darwin House, 12 Roger Street, WC1N 2JU, UK
Registered as a Charity: 264017 (England & Wales); SC039250 (Scotland)
Standard_article_template

Only bootstrap values of more than 50% was shown. *Reinekea marinisedimentorum* DSM 15388^{T} was used as an outgroup. GenBank accession numbers were provided after the species names. Bar, 0.01 substitutions per nucleotide position.

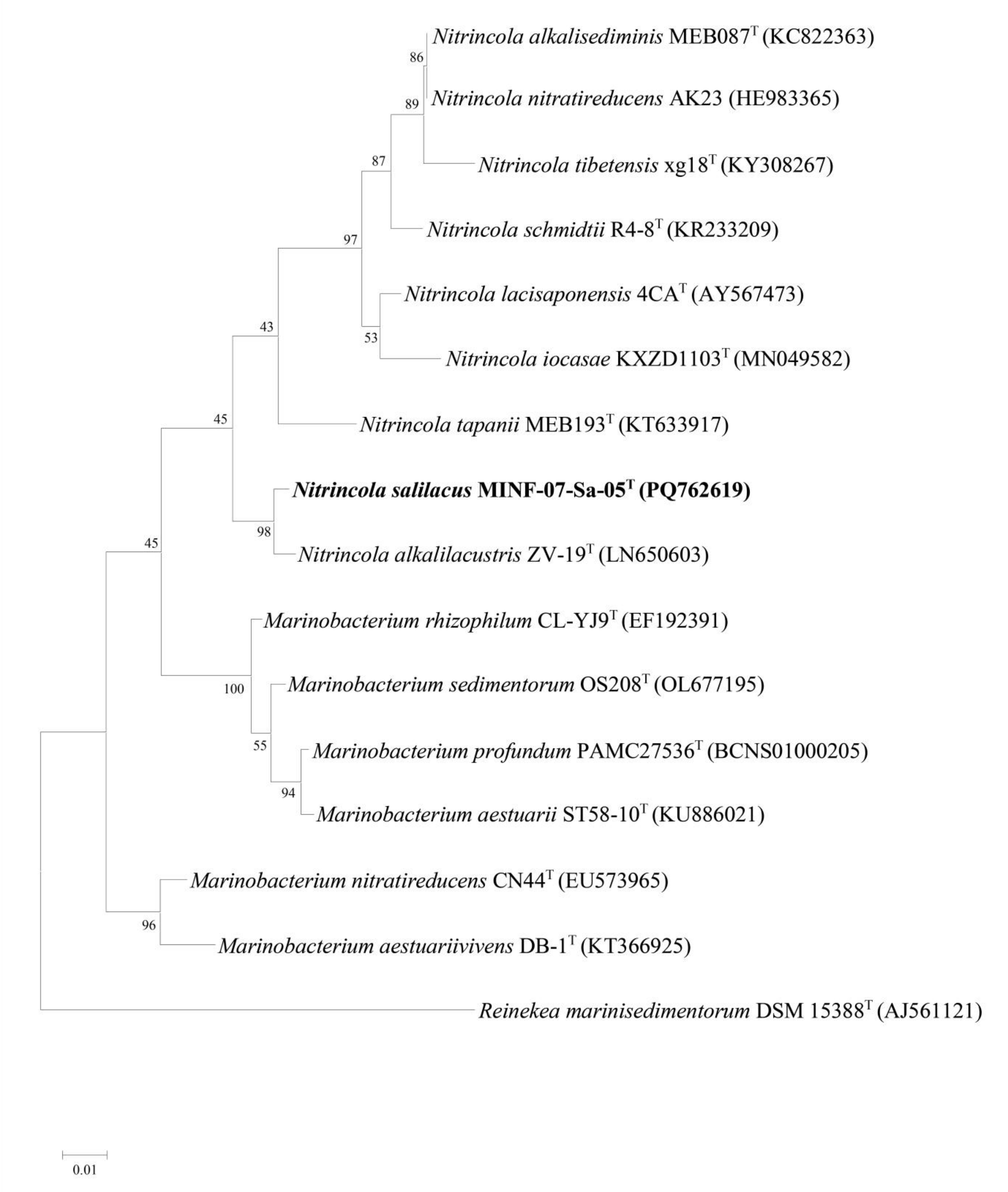

Fig. S2. Maximum-likelihood phylogenetic tree based on 16S rRNA gene sequences showing the relationships between strain MINF-07-Sa-05^{T} and other related taxa in the family of *Oceanospirillaceae*. Bootstrap values were expressed as a percentage of 1000 replications. Only bootstrap values of more than 50% were shown. *Reinekea marinisedimentorum* DSM 15388^{T} was used as an outgroup. GenBank accession numbers were provided after the species names. Bar, 0.01 substitutions per nucleotide position.

Table S1. Comparison of MINF-07-Sa-05^{T} and the other related strains of genus *Nitrincola* genomes by the 16S rRNA gene sequences similarity, Genome-to-Genome Distance Calculator (GGDC) and average nucleotide identity (ANI) analysis.

Closely related species	16s rRNA (%)	Genomic relatedness (%)	
		ANI	dDDH
N. alkalilacustris ZV-19^{T}	98.97	87.79	33.70
N. lacisaponensis 4CA^{T}	96.16	72.03	21.20
N. tapanii MEB193^{T}	95.96	71.03	21.10
N. iocasae KXZD1103^{T}	95.41	72.15	22.40
N. alkalisediminis MEB087^{T}	95.20	70.10	21.30
N. tibetensis xg18^{T}	95.07	69.76	22.20
N. schmidtii R4-8^{T}	94.86	69.92	20.40
N. nitratireducens AK23	95.53	69.82	20.80

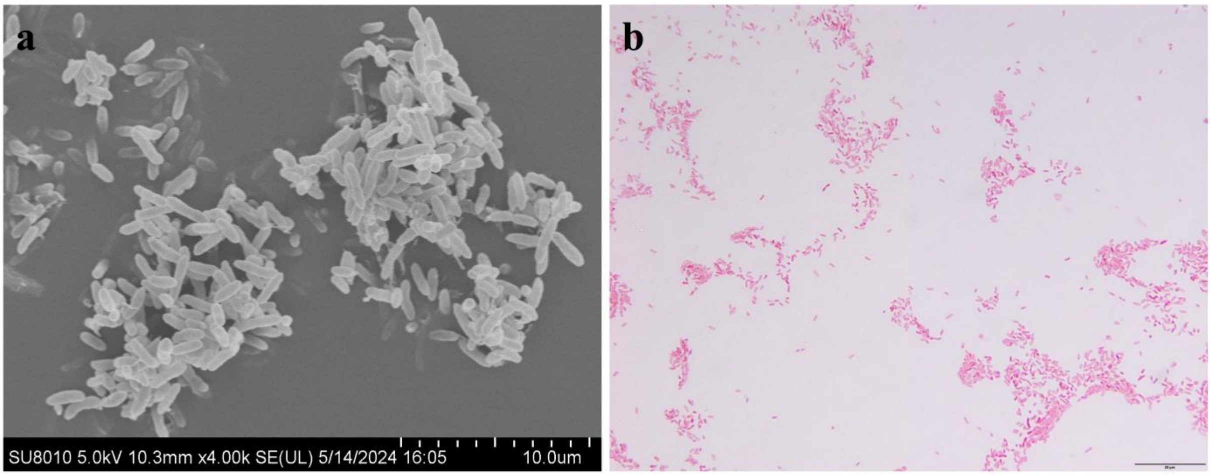

Fig. S3. (a) Scanning electron micrograph of strain MINF-07-Sa-05^{T}. Bar, 10.0 μm; (b) Gram -stain of cells. Bar, 20 μm.

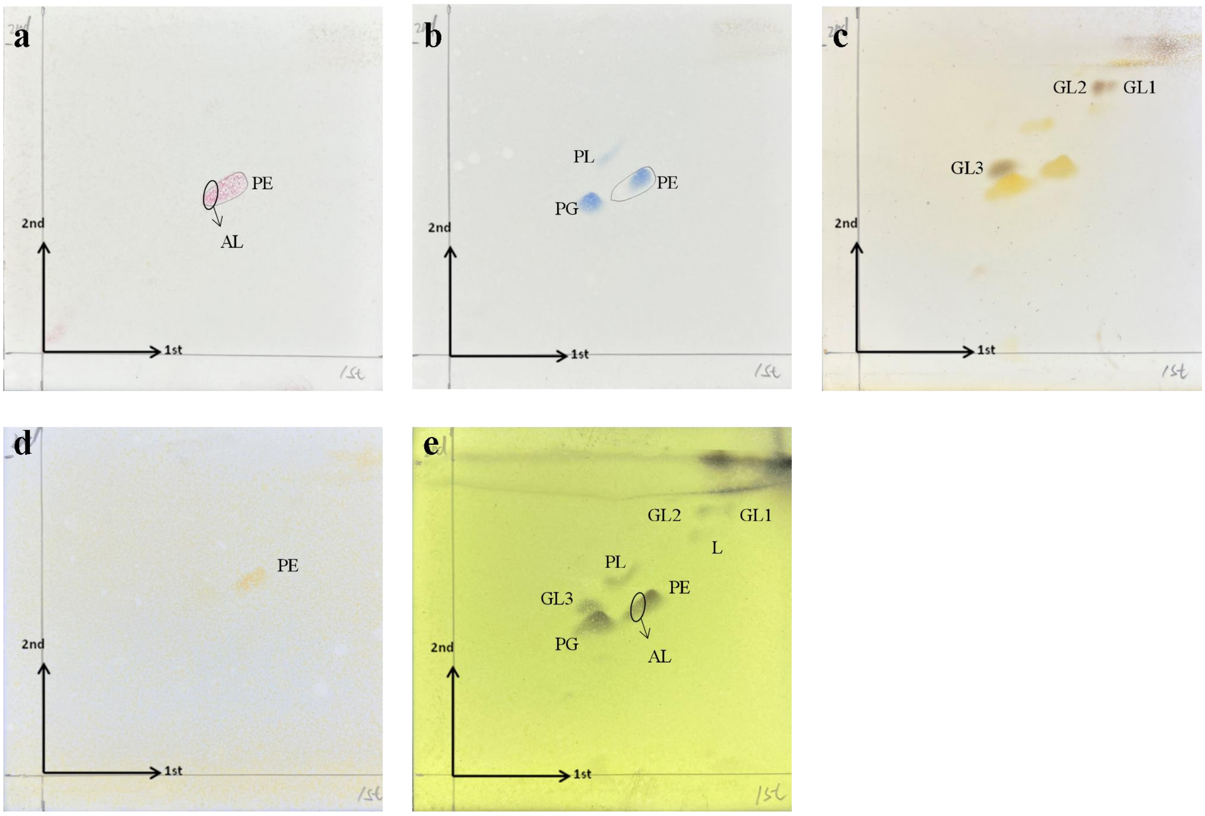

Fig. S4. Polar lipid profiles of strain MINF-07-Sa-05^T.
The extracted lipids were identified by spraying with ninhydrin (a), molybdenum blue (b), alpha-naphthol (c), Dragendorff's reagent (d), and molybdosphoric acid (e).
Chloroform/methanol/water (65:25:4, v/v/v) was used in the first direction, followed by chloroform/methanol/acetic acid/water (80:12:15:4, v/v/v/v) in the second direction
Abbreviations: PG, phosphatidylglycerol; PE, phosphatidylethanolamine; AL, unidentified aminolipid; PL, unidentified phospholipid; GL1-3, unidentified glycolipids; L, unidentified lipid.

Table S2. Genomic features of strain MINF-07-Sa-05^T.

Strain Name	MINF-07-Sa-05^T
Size(bp)	4817508
G+C content(bp)	2533853
Coding region(bp)	4363944
Total genes	4498
RNA genes	94
Protein-coding genes	4404
Protein coding genes with enzymes	1529
Genes assigned to COGs	3584
COG clusters	1876
Genes with signal petides	325
NR	4236
KEGG	3192
GO	2755
COG	3584
CAZy	377
Pfam	3836
Swiss-Prot	3262

CARD	298
VFDB	2

Table S3. The genes involved in nitrogen cycling based on annotation of genome of strain MINF-07-Sa-05[T].

Pathways	Gene	Annotation	Location of genes	
			Start	Stop
Assimilatory nitrogen Reduction	*nasA*	Assimilatory nitrate reductase catalytic subunit	504035	506749
$NO_3^- \rightarrow NO_2^-$	*nasB*	Assimilatory nitrate reductase electron transfer subunit	512319	513539
	NR	Nitrate reductase	890417	891847
$NO_2^- \rightarrow NH_4^+$	*nirA*	Ferredoxin-nitrite reductase	2876262	2877920
Dissimilatory nitrogen Reduction and Denitrification	*narG*	Nitrate reductase 1, alpha subunit	4401743	4405495
	narH	Nitrate reductase 1, beta subunit	4400187	4401746
	narJ	Nitrate reductase 1, delta subunit	4399440	4400174
	narI	Nitrate reductase 1, gamma subunit	4398745	4399422
$NO_3^- \rightarrow NO_2^-$	*narZ*	Nitrate reductase 2, alpha subunit	4401743	4405495
	narY	Nitrate reductase 2, beta subunit	4400187	4401746
	narW	Nitrate reductase 2, delta subunit	4399440	4400174
	narV	Nitrate reductase 2, gamma subunit	4398745	4399422
	napA	Periplasmic nitrate reductase subunit	1173745	1176249
	napB	Periplasmic nitrate reductase electron transfer subunit	1173243	1173731
	napC	Nitrate reductase cytochrome c-type periplasmic	1172656	1173246
$NO_2^- \rightarrow NH_4^+$	*nirB*	Nitrite reductase (NADH) large subunit	512319	513539
	nirD	Nitrite reductase (NADH) small subunit	516084	516434
$NO_2^- \rightarrow NO$	*nirK*	Nitrite reductase (NO-forming)	363067	363369
Nitrification $NO_2^- \rightarrow NO_3^-$	*nxrA*	Nitrite oxidoreductase, alpha subunit	4401743	4405495
	nxrB	Nitrite oxidoreductase, beta subunit	4400187	4401746

Table S4. The genes involved in sulphur cycling based on annotation of genome of strain MINF-07-Sa-05[T].

Pathways	Gene	Annotation	Location of genes	
			Start	Stop
Assimilatory sulfate reduction	*cysC*	Adenylylsulfate kinase	2879288	2880979
	cysD	Sulfate adenylyltransferase subunit 2	2878314	2879231
	cysH	Phosphoadenosine phosphosulfate reductase	2875130	2875753
	cysI	Sulfite reductase (NADPH) hemoprotein beta-component	2876262	2877920
	cysJ	Sulfite reductase (NADPH) flavoprotein alpha-component	182759	184612
	cysN	Sulfate adenylyltransferase subunit 1	2879288	2880979
	cysNC	Bifunctional enzyme	2879288	2880979
	cysQ	3'(2'), 5'-bisphosphate nucleotidase	224600	225430

Dissimilatory sulfur reduction and oxidation	*aprA*	Adenylylsulfate reductase, subunit A	1658654	1666669
	dsrC	Dissimilatory sulfite reductase related protein	2315875	2316210
	dsrE	Sulfurtransferase	2316864	2317256
	dsrF	Intracellular sulfur oxidation protein	2316497	2316862
	dsrH	Intracellular sulfur oxidation protein	2316213	2316497
Sulfur reduction	*hydA*	Sulfhydrogenase subunit alpha	721625	723070
	hydG	Sulfhydrogenase subunit gamma (sulfur reductase)	768923	770716
SOX systems	*soxA*	L-cysteine S-thiosulfotransferase	4452508	4455534
	soxB	S-sulfosulfanyl-L-cysteine sulfohydrolase	4455855	4457105
	soxD	S-disulfanyl-L-cysteine oxidoreductase	4455531	4455839
	soxY	Sulfur-oxidizing protein	1266816	1267631
	soxZ	Sulfur-oxidizing protein	3100094	3100402
Sulfur oxidation	*fccA*	Cytochrome subunit of sulfide dehydrogenase	1461731	1462048
	fccB	Sulfide dehydrogenase (flavocytochrome c) flavoprotein chain	1462059	1463381
	glpE	Thiosulfate sulfurtransferase	1071192	1071509
	sqr	Sulfide:quinone oxidoreductase	2440923	2442308
	sseA	Thiosulfate sulfurtransferase	3462321	3463166
Organic sulfur transformation	*acuI*	Acrylyl-CoA reductase	1276888	1277886
	betB	NAD/NADP-dependent betaine aldehyde dehydrogenase	4296870	4298351
	betC	Choline-sulfatase	2482655	2484178
	comA	Phosphosulfolactate synthase	1898258	1900516
	comC	L-sulfolactate dehydrogenase	4753949	4754944
	ddhC	Dimethylsulfide dehydrogenase subunit gamma	3594453	3595631
	dmdA	Dimethylsulfoniopropionate demethylase	4052702	4053853
	dmdC	3-(methylthio)propanoyl-CoA dehydrogenase	612356	614089
	dmdD	(methylthio)acryloyl-CoA hydratase	609532	610296
	gdh	Glutamate dehydrogenase ($NADP^+$)	692558	697378
	mdh	Malate dehydrogenase	2313011	2314111
	prpE	Propionate-CoA ligase	1721583	1723478
	pta	Phosphate acetyltransferase	960717	961673
Link between inorganic and organic sulfur transformation	*cysE*	Serine O-acetyltransferase	3313461	3314258
	cysK	Cysteine synthase	4058042	4058941
	cysM	Cysteine synthase	4058042	4058941
	metX	Homoserine O-acetyltransferase/O-succinyltransferase	4506671	4507843
	metY	O-acetyl-L-homoserine sulfhydrylase	344288	345559
	metZ	O-succinylhomoserine sulfhydrylase	2125667	2126884
Others	*cysA*	Sulfate/thiosulfate import ATP-binding protein	3327323	3328441
	cysZ	Sulfate transporter	2808109	2808858
	ssuA	Sulfonate transport system substrate-binding protein	3272277	3273326
	tusA	Sulfur carrier protein	1751738	1751974
	tusB	tRNA 2-thiouridine synthesizing protein B	2316213	2316497
	tusC	tRNA 2-thiouridine synthesizing protein C	2316497	2316862
	tusD	Sulfurtransferase	2316864	2317256

	tusE	Sulfurtransferase	2315875	2316210

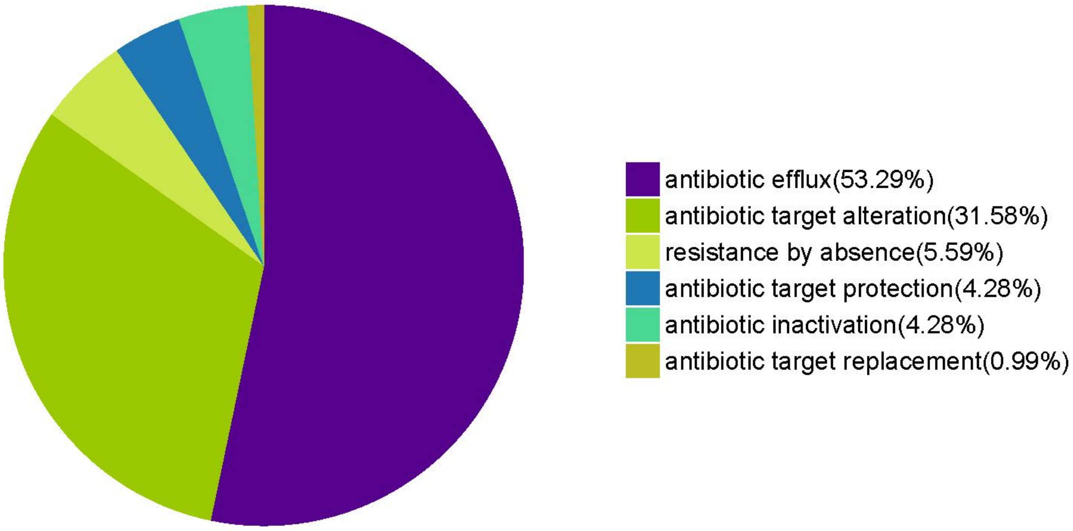

Fig. S5. Distribution of antibiotic resistance genes in strain MINF-07-Sa-05^T.

Table S5. Antibiotic resistance gene prediction of strain MINF-07-Sa-05^T.

Pathways	Gene	Annotation	Location of genes	
			Start	Stop
Macrolide antibiotic	*macB*	MacB was an ATP-binding cassette (ABC) transporter that exported macrolides with 14- or 15- membered lactones	69218	70357
	mtrA	Transcriptional activator of the MtrCDE multidrug efflux pump	1357489	1358172
Fluoroquinolone antibiotic	*evgS*	Sensor protein that phosphorylated the regulatory protein EvgA.	1879612	1881462
	patA	PatA was an ABC transporter interacted with PatB to confer fluoroquinolone resistance	2377275	2378489
Tetracycline antibiotic	*tetA*	Tetracycline efflux pump	2502417	2504189
	tetT	Ribosomal protection protein	4063706	4065505
Triclosan	*fabG*	3-oxoacyl-acyl carrier protein reductase involved in lipid metabolism and fatty acid biosynthesis	4344364	4345092
	opmH	Outer membrane efflux protein required for triclosan-specific efflux pump function	2796399	2797709
Aminoglycoside antibiotic	*pvrR*	A response regulator that controled the conversion between antibiotic-resistant and antibiotic-susceptible forms	2916100	2918337
	AAC(3)-Id	An aminoglycoside acetyltransferase	1471020	1471484

Table S6. General features of bacterial genomes for comparative genomic analysis.

Strain Name	GenBank	Size (Mb)	GC%	Level	Contigs	N50 (kb)	L50	No. of Genes	No. of Proteins	Isolation
N. salilacus MINF-07-Sa-05^{T}	GCA_046271865.1	4.82	52.6	Complete	1	4817.5	1	4,435	4,324	a salt lake
N. alkalilacustris ZV-19^{T}	GCA_008973755.1	4.06	54.5	Contig	25	492.1	4	3,725	3,641	water of soda pans
N. lacisaponensis 4CA^{T}	GCA_000691225.1	3.41	52.1	Contig	43	336.5	3	3,192	3,112	shore of Soap Lake
N. tapanii MEB193^{T}	GCA_008368715.1	2.79	50.8	Contig	19	282.3	4	2,624	2,548	a soda lake
N. iocasae KXZD1103^{T}	GCA_008727795.1	4.17	50.1	Complete	2	4173.0	1	3,889	3,788	a cold seep field
N. alkalisediminis MEB087^{T}	GCA_009821115.1	3.97	49.3	Scaffold	36	270.6	5	3,685	3,587	an alkaline saline lake
N. tibetensis xg18^{T}	GCA_003284585.1	4.00	46.1	Contig	54	255.7	5	3,678	3,588	an alkaline lake
N. schmidtii R4-8^{T}	GCA_008973715.1	3.14	45.8	Scaffold	35	204.9	6	2,936	2,860	water of soda pans
N. nitratireducens AK23	GCA_000585235.1	4.03	46.8	Contig	68	133.9	9	3,962	3,659	a haloalkaline lake

Company Limited by Guarantee l Registered in England No. 1039582 l Registered Office Charles Darwin House, 12 Roger Street, WC1N 2JU, UK
Registered as a Charity: 264017 (England & Wales); SC039250 (Scotland)
Standard_article_template
